# Gut microbiota-derived EPA alleviates neuroinflammation associated with white matter injury by influencing H3K9ac/BDNF/TrkB pathway

**DOI:** 10.3389/fmicb.2026.1711114

**Published:** 2026-03-05

**Authors:** Yuqian Wang, Yajun Zhang, Yifan Cui, Jing Zhu, Chan Wang, Shanshan Wang, Xuwu Xiao, Liu Yang

**Affiliations:** 1 Department of Pediatrics, The Second Hospital of Dalian Medical University, Dalian, Liaoning, China; 2 Department of Anesthesia, Dalian Women and Children’s Medical Group, Dalian, Liaoning, China; 3 Department of Pediatrics, Dalian Women and Children’s Medical Group, Dalian, Liaoning, China

**Keywords:** EPA, fecal microbiota transplantation, gut microbiota, histone acetylation, microbiota-gut-brain axis, neuroinflammation, white matter injury

## Abstract

**Background:**

The objective of our investigation was to explore the features of gut microbiota dysbiosis and the concentrations of gut metabolites in relation to white matter injury (WMI). Furthermore, we sought to evaluate the influence of gut dysbiosis on neuroinflammation in WMI via intestinal metabolites, and its contribution to pathogenesis.

**Methods:**

A cerebral hypoxia-ischemia-induced WMI model was established in 3-day-old Sprague–Dawley rats. Liquid chromatography-mass spectrometry/gas chromatography–mass spectrometry analyses and 16S rRNA gene sequencing were undertaken to ascertain WMI biomarkers. Mechanistic experiments were used to analyse activation of the H3K9ac/BDNF/TrkB pathway and neuroinflammation.

**Results:**

The analysis of 16S rRNA sequencing disclosed gut microbiota dysbiosis in WMI rats, quantified using linear discriminant analysis effect size. Overall, 341 differentially expressed metabolic markers between the WMI and Sham groups were discovered. The Kyoto Encyclopedia of Genes and Genomes network enhancement evaluation revealed significant downregulation of 20 metabolic processes in the WMI group, which is strongly related to changes in fecal microbial metabolites, and the synthesis process of unsaturated fatty acids was the most significant. Gut microbiota dysbiosis may influence WMI by downregulating metabolites such as eicosapentaenoic acid (EPA). Fecal microbiota transplantation increased EPA concentration in the brain tissue of WMI rats. Gut microbiota-derived EPA promoted H3K9ac and BDNF/TrkB expression and inhibited the transcription of pro-inflammatory TNF-*α* and IL-1β molecules. These EPA-mediated effects were reversed by TrkB inhibition.

**Conclusion:**

WMI induces gut dysbiosis involving down-regulation of unsaturated fatty acid synthesis. Fecal microbiota transplantation leads to increased levels of EPA. Gut microbiota-derived EPA increases levels of acetylated histone H3K9ac, causes activation of the BDNF/TrkB pathway, reduces neuroinflammation, and improves WMI-associated myelination disorders. It provides a basis for targeted treatment of white matter injury in the future.

## Highlights

- Key Message: Neonatal WMI is associated with gut microbiota dysbiosis and EPA depletion, contributing to neuroinflammation and impaired myelination.
- Novel Contribution: First to link gut microbiota, metabolomics, and epigenetics (H3K9ac/BDNF/TrkB) in WMI, identifying EPA as a neuroprotective metabolite.
- Mechanistic Insight: EPA promotes myelination via histone acetylation and BDNF/TrkB pathway activation.
- Clinical Implication: FMT and microbial metabolites like EPA may reduce neuroinflammation and aid myelination in preterm infants.
- Scientific Impact: Offers new microbiota-based strategies for understanding and treating neonatal brain injury.

## 1 Introduction

WMI represents a specific type of damage to the brain that may result in long-term adverse neurological outcomes, such as cerebral palsy, mental impairment, intellectual disability, and neurobehavioral disorders in premature infants (Volpe, 2008; Yue et al., 2018; Cooper et al., 2017). Perinatal infections, inflammation, and hypoxia-ischemia contribute to the pathophysiological processes underlying WMI. The primary pathological process in WMI is impaired myelin development, and effective targeted therapies are still lacking.

The gut microbiota, termed the ‘second genome’, is the body’s largest ecosystem, comprising trillions of microorganisms (Wan et al., 2020). It plays a crucial role in maintaining immune homeostasis and promoting overall health. The microbiota-gut-brain axis (MGBA) establishes a two-way connection between the gastrointestinal system and the brain, facilitating the integration of nerve, hormone, and immune signaling. The MGBA is therefore instrumental in regulating the effects of gut microbiota and its metabolites in brain functions (Filpa et al., 2016; Sánchez et al., 2017). Normal gut microbiota colonization coincides with the peak development of oligodendrocytes and formation of brain myelin (Ahmed et al., 2021). The gut microbiota exerts a profound impact on cognitive performance and human behavior through the intricate microbiota-gut-brain axis (Feng et al., 2022). Our prior Mendelian Randomization investigation identified causal links between particular gut microbiota and detrimental neurological development in babies born prematurely (Wang et al., 2024). Preterm babies are affected by multiple factors that delay colonization of the gut microbiota (Beghetti et al., 2022). An impaired MGBA may trigger or aggravate cerebral trauma in premature newborns.

There is a notable deficiency in research exploring the relationship between intestinal microbiota and cognitive function in premature newborns (Ruggiero, 2016; Oliva-Hemker et al., 2023). Furthermore, the composition of gut microbiota, its mechanisms, and potential microbial markers of WMI remain unclear. Examining the connection between gut microbiota as well as the framework and operational aspects of the brain in premature newborn are of paramount importance. Fecal microbiota transplantation (FMT) positively regulates microbial disorders associated with neurological diseases, facilitating nerve regeneration in murine models with spinal cord injuries through the inhibition of blood-spinal cord barrier compromise, enhancement of neurotrophic factor expression, mitigation of spinal cord ischemia, and the promotion of neuronal viability. Fresh sterile FMT effectively reduces inflammation, oxidative injury and histopathological alterations in both the intestine and brain, promoting it as an intervention to minimise brain injury in premature infants (Prado et al., 2022). Sodium butyrate, an intestinal metabolite, alleviates cognitive and memory impairment caused by lead exposure via the ACSS2/H3K9ac/BDNF pathway (Li et al., 2024). The gut microbiome significantly influences the pathophysiology and cognitive deficits related to Alzheimer’s disease through the regulation of neuroinflammation linked to polyunsaturated fatty acids (PUFA) (Chen et al., 2022). This evidence suggests that modulating gut microbiota dysbiosis could serve a neuroprotective function through the regulation of intestinal metabolites. Epigenetic modifications modulate the neural repair of WMI caused by hypoxia-ischaemia (Zhang et al., 2024) in a conserved manner linked to nervous system repair. Prevalent epigenetic processes encompass DNA methylation, histone alterations, and chromosomal remodeling (Ogino et al., 2013). Histone acetylation is fundamental to the control of gene expression. Acetylation and deacetylation are chiefly governed by histone acetyltransferases and histone deacetylases, which facilitate the relaxation of chromatin architecture, enhance gene transcription, and direct tissue development and regeneration. Inhibiting HDAC activity increases histone acetylation levels, thereby increasing oligodendrocyte populations, promoting white matter repair, enhancing axonal myelination, and aiding striatal neuron regeneration (Chen et al., 2012).

Given this context, this investigation utilized 16S rRNA gene sequencing in conjunction with liquid chromatography-mass spectrometry (LC–MS) and gas chromatography–mass spectrometry (GC–MS) to compare alterations in gut microbiota and metabolites between the WMI and Sham models. Further, we investigated how intestinal metabolites can protect the nervous system through neuroinflammation and epigenetic regulation, thereby elucidating the mechanistic role of the MGBA in WMI.

## 2 Materials and procedures

### 2.1 Experiments involving animals

Animal experiments, utilizing neonatal Sprague–Dawley rodents sourced from SPF (Beijing) Biotechnology company, obtained consent from the Animal Research Ethics Committee of Dalian Medical University. The rats had unrestricted access to food and water and were kept in a 12-h light/dark cycle.

#### 2.1.1 Model establishment

Cerebral WMI was induced in neonatal rats, following the procedure previously employed by our research team (Vannucci et al., 1999; Cheng et al., 2015). Male and female SD rats that were 3 days old were carefully placed in a prone posture on the surgical table after being sedated with isoflurane inhalation. SD rats were anesthetized using 3–5% isoflurane inhalation and 1–2% isoflurane for maintenance. The left common carotid artery was carefully revealed through the use of an anatomical microscope. Within the WMI category, the left common carotid artery underwent permanently ligated at both ends using sterilized needle ligatures, after which the segment located between the ligations was excised. The cut made during surgery was carefully closed with stitches. The duration of the surgical operation spanned approximately 8–10 min. After recovering from anaesthesia, rats were brought back together with their dams for a duration of 1 h, prior to being situated in a minimal oxygen chamber kept at 37 °C. Over 2.5 h, a gas combination of 8% oxygen and 92% nitrogen was continuously fed at 2 L/min to the low-oxygen chamber, resulting in an oxygen level of 8%. The Sham group experienced a dissection of the left common carotid artery, conducted absent of binding or being exposed to hypoxic conditions.

#### 2.1.2 Sample collection

SD rats were modeled at 3 days of age, and specimens were collected 7 days after modeling. SD rats were anesthetized with 5% isoflurane inhalation to expose the heart. After anesthesia, some rats were perfused with normal saline through the heart. The lateral hemisphere of the brain was separated and dissected on ice to isolate the cortex. The brain tissue around the lateral ventricle was placed in an EP tube and stored at −80 °C for molecular biology analyses (Western blot, RT-PCR, ELISA). The remaining rats were perfused with 4% paraformaldehyde through the heart, and the brain tissue was taken out and placed in 4% paraformaldehyde for histological examination (Hematoxylin and eosin staining, Immunohistochemical staining).

#### 2.1.3 Animal experiment 1:16S rRNA sequencing and LC–MS/GC–MS evaluation

Three-day-old SD rats were selected at random to either the Sham or WMI category, with fecal samples collected via sterile cotton swabs 1 week following the establishment of the model (age 10 days). Each EP tube contained approximately 0.1 g of feces, equivalent to about five to six pellets. The specimens were meticulously encapsulated. Appropriately labeled and preserved at −80 °C in anticipation of genetic evaluation.

#### 2.1.4 Animal experiment 2: FMT

The 3-day-old SD rats were allocated at random to the Sham, WMI, or WMI + FMT categories. One week after the model was established (age 10 days), brain tissue samples were collected for subsequent analysis. To prepare the FMT bacterial solution, fresh feces from healthy mice of the same age were collected daily within 3 h. Fecal supernatant was obtained by homogenizing 1 g of fresh stool in 10 mL of sterile PBS, followed by centrifugation at 2000 rpm for 5 min. This supernatant was then administered through FMT at a dosage of 30 μL per gram of the weight of the body via rectal administration (Liu et al., 2020). To ensure efficient administration, the tube was introduced into the mouse intestine to a depth of 1–3.5 cm. Following the injection, the mouse was positioned vertically with its head directed downward for a duration of 1 min to ensure retention of the injectant. FMT treatment was administered for 3 consecutive days from the second day after successful modelling.

#### 2.1.5 Animal experiment 3: mechanistic exploration

The 3-day-old SD rats were randomly assigned to the Sham, WMI, WMI + EPA, or WMI + EPA + ANA-12 groups. ANA-12 is an inhibitor of TrkB. One week after model establishment (age 10 days) brain tissue samples were collected for subsequent analysis. The WMI + EPA and WMI + EPA + ANA-12 groups received intraperitoneal injections of EPA (0.25 mg, AbMole, China) for a duration of 3 days, commencing on the second day following the successful completion of the modelling process (Jiao et al., 2021). The WMI + EPA + ANA-12 group received an intraperitoneal injection of ANA-12 (dissolved in corn oil with 1% dimethyl sulfoxide, 0.5 mg/kg via intraperitoneal injection), over 3 days, starting from the second day after successful modelling.

### 2.2 Extraction of DNA and amplification through PCR

#### 2.2.1 Isolation and amplification of DNA

Genomic DNA was isolated utilizing a MagPureStool/Soil DNA KF Kit in accordance with the manufacturer’s guidelines. The measurement of DNA concentration and authenticity was conducted utilizing the NanoDrop 2000 alongside agarose gel electrophoresis. DNA was collected and kept at −20 °C and used a base for PCR amplification of bacterial 16S rRNA genes with barcoded primers and Takara Ex Taq. In the examination of microbial variations, the V3-V4 (or V4-V5) variable areas of 16S rRNA genes were amplified using universal primers 343F and 798R (Nossa et al., 2010) for the V3-V4 areas.

#### 2.2.2 Sequencing and construction of library

The evaluation of the Amplicon was conducted via visualization via gel electrophoresis. The purification of PCR results was carried out utilizing Agencourt AMPure XP beads from Beckman Coulter Co., USA, followed by measurement with a Qubit dsDNA assay kit. Concentrations were meticulously calibrated for sequencing, executed utilizing the Illumina NovaSeq system-6000, which encompassed two paired-end read cycles, each consisting of 250 bases. Illumina Inc., located in San Diego, California, and OE Biotech Company, based in China.

#### 2.2.3 Analysis in bioinformatics

The library sequencing and the ensuing analysis of information were carried out by OE Biotech Co., Ltd., based in China. The sequencing data in its unprocessed form was provided in the FASTQ structure. Paired-end reads were pre-processed through the application of Cutadapt program, which was utilized to find and eliminate adapters. Subsequent to the cutting procedure, paired-end reads were employed to discard inferior sequences, perform denoising, merge, and detect as well as eliminate chimera reads using DADA2 (Callahan et al., 2016) with the standard factors of QIIME2 (Bolyen et al., 2019). Two samples were excluded due to an insufficient number of sequencing reads (< 1,000). The final analysis thus included 5 samples in the WMI group and 5 samples in the Sham group. Ultimately, the program produced the tables detailing representativeness and ASV abundance. The representative reads for each ASV were selected utilizing the QIIME2 program. All representative reads underwent annotation and were subjected to BLAST analysis against the Silva database utilizing the q2-feature-classifier with the standard configurations. The application of QIIME2 program facilitated the analyses of alpha and beta diversity. The estimation of microbial diversity in the specimens was conducted through alpha diversity parameters, incorporating the Chao1 index (Chao and Bunge, 2002), Ace index, Observed_species index, and Shannon index (Hill et al., 2003). Based on the data distribution and homogeneity of variance, differences in alpha diversity indices and taxonomic abundances were assessed using either parametric tests (Student’s *t*-test or ANOVA) or their non-parametric counterparts (Wilcoxon rank-sum test or Kruskal-Wallis test). When statistically significant differences were detected (*p* < 0.05), post-hoc pairwise comparisons were conducted. The weighted UniFrac distance matrix generated using the R package facilitated weighted UniFrac Principal Coordinates Analysis, while the Bray-Curtis distance matrix, also derived from the R program, was employed for Bray-Curtis non-metric multidimensional scaling to determine beta diversity. To validate significant inter-group differences, a modified multivariate analysis of variance (PERMANOVA) was performed using the adonis2 function with 999 permutations, supplemented by similarity analysis (ANOSIM). Additionally, the Linear Discriminant Analysis Effect Size (LEfSe) method was employed to identify potential biomarker classification units. This approach first applied the Kruskal–Wallis test (*p* < 0.05) to detect features with significant differences across groups. Subsequently, Linear Discriminant Analysis (LDA) was used to estimate the effect size, with features attaining an LDA score > 2.0 retained as significant discriminant factors. All statistical analyses and graphical outputs, including box plots for alpha diversity and bar plots for LDA biomarkers, were generated within the R statistical environment.

### 2.3 Metabolite extraction and quality control sample

Groups for non-targeted metabolite analysis contained five fecal samples (Liang et al., 2020; Davidsen et al., 2020; Arndt et al., 2020; Viant et al., 2017). Fecal samples, precisely measured at 15 mg, were carefully positioned within a 1.5 mL centrifuge tube. Following this, 2 diminutive steel spheres and 300 μL of a methanol–water solution (in a 4:1 volume ratio, incorporating a mixed internal standard at a concentration of 4 μg/mL) were introduced. Following a pre-cooling phase at −40 °C for a duration of 2 min, the specimens underwent grinding at a frequency of 60 Hz for 2 min. For 10 min, ultrasonic extraction was carried out in an ice bath, subsequently, an incubation at −40 °C for 2 h was conducted; centrifugation was conducted for 20 min at 13,000 rpm and 4 °C. A syringe was employed to withdraw 150 μL of supernatant, that underwent a filtration process utilizing a 0.22 μm organic phase pinhole filter, after which it was transferred to an LC injection vial for storage at −80 °C, pending subsequent LC–MS analysis. Concurrently, 100 μL from the supernatant was carefully put into a glass derivative vial, followed by the specimen undergoing a drying process utilizing a centrifugal concentrator dryer. Following this, 80 μL of a methoxyamine hydrochloride pyridine solution was added to the glass vial, and the oximation process was carried out in an incubator at 37 °C for a period of 60 min. Upon the removal of the specimen, 50 μL of BSTFA derivatization reagent was introduced, accompanied by 20 μL of n-hexane, in addition to 10 μL of a mixture of 10 internal standards, all solubilized in chloroform. The combination underwent a reaction at 70 °C for 60 min. Upon extraction, the specimens were permitted to reach equilibrium at ambient temperature for 30 min before undergoing GC–MS metabolomics evaluation. An analytical examination was conducted utilizing an Agilent 7890B gas chromatography system (Agilent Technologies, USA) alongside a Waters ACQUITY UPLC I-Class plus/Thermo QE HF liquid chromatography system. The injector temperature was maintained at 230 °C. The injection volume was established at 1 μL employing the splitless mode. The energy of the collision was established at 70 eV. Data from mass spectrometry were obtained in full-scan mode, covering the range of m/z 50 to 500. The data underwent preliminary treatment utilizing Progenesis QI v3.0. The Human Metabolome Database, Lipidmaps, METLIN repository, and LuMet-Animal 3.0 local records were employed for the purposes of identification and analysis. For targeted metabolic analysis, each group contained 3 brain samples (Liang et al., 2020; Davidsen et al., 2020; Arndt et al., 2020; Viant et al., 2017). The application of GC–MS/MS analysis facilitated both quantitative as well as qualitative detection of targeted fatty acids. A total of 50 mg of brain samples were meticulously weighed and subsequently extracted using 150 μL of a methanol solution, 200 μL of a methyl tert-butyl ether solution, and 50 μL of a 36% phosphoric acid solution. The solution underwent vortexing for a duration of 3 min, followed by centrifugation at a temperature of 4 °C and a speed of 12,000 rpm for 5 min. Following this, 200 μL of the supernatant was meticulously retrieved, subjected to drying via a nitrogen stream, and subsequently resuspended in 300 μL of a 15% boron trifluoride solution in methanol. The solution underwent vortexing for a duration of 3 min, subsequently subjected to an incubation period lasting 30 min at 60 °C. After reaching room temperature, 500 μL of n-hexane and 200 μL of saturated sodium chloride were introduced. After 3-min vortexing, the mixture was centrifuged at 4 °C at 12,000 rpm for 5 min. Next, 100 μL of the n-hexane layer solution was collected for online evaluation. Using triple quadrupole mass spectrometry’s MRM mode, data from many specimens were collected and all chromatographic peak regions were combined to quantify metabolites. Multivariate statistical analysis was employed to analyze the metabolic profiles and identify differentially abundant metabolites between experimental groups. Specifically, Orthogonal Partial Least Squares-Discriminant Analysis (OPLS-DA) was performed using pre-processed and normalized metabolomic data to maximize the separation between groups while filtering out non-correlated variation (orthogonal signal). The robustness and predictive ability of the OPLS-DA model were rigorously validated through a seven-round interactive cross-validation procedure. Model quality was assessed using parameters R^2^Y (goodness of fit) and Q^2^ (goodness of prediction), where a Q^2^ value greater than 0.5 typically indicates a model with reliable predictive power and low risk of overfitting. To identify metabolites that contributed most significantly to the observed inter-group separation, a combination of criteria was applied. Metabolites with a Variable Importance in Projection (VIP) score > 1.0 were considered influential to the model. This statistical significance was further refined by univariate analysis (Student’s or Wilcoxon rank-sum test, *p* < 0.05), ensuring that the selected metabolites exhibited both high group-discriminatory power and statistically significant abundance changes. Metabolites satisfying both VIP > 1 and *p* < 0.05 were defined as differentially abundant metabolites (DAMs). Subsequently, these identified DAMs were subjected to functional enrichment and pathway analysis to elucidate the underlying disturbed biological processes. The metabolites were mapped to their known biochemical pathways using the Kyoto Encyclopedia of Genes and Genomes (KEGG) database. Metabolite set enrichment analysis was performed to identify pathways that were significantly enriched with DAMs, with significance judged by a hypergeometric test (*p* < 0.05). Additionally, pathway impact analysis was conducted by integrating pathway topology to highlight key metabolic routes most perturbed in the experimental context, often visualized via pathway impact plots.

### 2.4 Hematoxylin and eosin staining

Brain tissue was preserved in 4% paraformaldehyde and subsequently stained with hematoxylin and eosin (HE) to facilitate a histopathological evaluation of alterations in tissue of the brain observed under a light microscope.

### 2.5 Immunohistochemical staining

Immunohistochemical staining for myelin basic protein (MBP) was performed on brain tissue samples fixed in 4% paraformaldehyde. Following fixation, tissues were embedded in paraffin and sectioned at a thickness of 4–5 μm. After deparaffinization and rehydration, antigen retrieval was conducted using citrate buffer. The sections were then incubated overnight at 4 °C with a monoclonal anti-MBP antibody (1:200; BF8010). Subsequent staining was carried out using an appropriate HRP-conjugated secondary antibody, and antigen–antibody binding was visualized with DAB. MBP-positive areas were quantitatively evaluated based on staining intensity using ImageJ software.

### 2.6 Western blot analysis

The brain tissue for preparing the protein was selected from the periventricular area after stripping the cortex. Phosphatase inhibitors from Beyotime, China, were added to RIPA buffer for lysis. Solarbio kits were used to acquire nuclear proteins and BCA assay kits to quantify protein levels. Proteins (50 μg) were electrophoretically separated using SDS-PAGE (4–20%) and transferred to PVDF membranes. After a 2-h blocking step with 5% skim milk, membranes were incubated overnight at 4 °C with primary antibodies. The following primary antibodies were: rabbit anti-MBP monoclonal antibodies (1:3000; BF8010; Affinity Biosciences), rabbit anti-BDNF monoclonal antibodies (1:5000; DF6387; Affinity Biosciences), rabbit anti-phospho-.

TrkB polyclonal antibodies (1:5000; AF3461; Affinity Biosciences), rabbit anti-AH3K9 monoclonal antibodies (1:5000; DF6937; Affinity Biosciences), rabbit anti-*β*-tubulin polyclonal antibodies (1:5000; Proteintech). This was followed with Servicebio secondary antibodies and Beyotime ECL substrates for visualization. ImageJ was used to quantify band intensities and standardize them to β-actin.

### 2.7 Real-time polymerase chain reaction

RT-PCR was used to assess TNF-*α* and IL-β gene expressions in the periventricular brain area that was blocked. To get RNA samples, the left midbrain segment was pulverized, homogenized, and centrifuged at 4 °C for 15 min at 4,000 rpm. Takara Bio in China, supplied the TRIzol reagent for total RNA extraction. RT-PCR was used to assess TNF-*α* and IL-β gene expressions in the periventricular brain area that was blocked. To prepare RNA samples, the left midbrain segment was pulverized, homogenized, and centrifuged at 4 °C at 4,000 rpm for 15 min. Takara Bio in China, supplied the TRIzol reagent for total RNA extraction. The primer sequences utilized in this study were specifically designed by the Shanghai Biotechnology Service company, located in China.

TNF-α forward 5’-GGTGATCGGTCCCAACAAGG-3′ and reverse 5’-CCTCCCAGGTACATGGGCTC-3’; IL-1β forward 5’-CCCTGAACTCAACTGTGAAATAGCA-3′ and reverse 5’-CCCAAGTCAAGGGCTTGGAA-3′.

### 2.8 Enzyme-linked immunosorbent assay

The inflammatory agents present in the ligated region of the periventricular brain tissue were assessed through the application of ELISA techniques. The brain tissue was subjected to the addition of PBS, followed by homogenization and centrifugation at 3500 rpm for a duration of 15 min to isolate the supernatant. The concentrations of IL-1β (JL18442; JONLNBIO, Shanghai, China) and TNF-α (JL13202; JONLNBIO, Shanghai, China) were quantified via ELISA, with optical density assessed at 450 nm utilizing a microplate reader (Synergy H4; Biotek).

### 2.9 Statistical analyses

All statistical analyses were performed using GraphPad Prism (version 10.1) and R software (version 4.3.2). Data are presented as the mean ± standard error of the mean (SEM), and Statistical significance was set at *p* < 0.05. For comparisons between two groups, unpaired *t*-tests were used, or the Mann–Whitney U test was employed for nonparametric data such as alpha-diversity. Comparisons among three or more groups were performed using one-way ANOVA with Tukey’s post-hoc test for parametric data or the Kruskal-Wallis test with Dunn’s post-hoc test for non-parametric data. Differences in beta-diversity were assessed using PERMANOVA. Differentially abundant microbial taxa were identified using Linear Discriminant Analysis Effect Size (LEfSe) (LDA score > 2.0, *p* < 0.05). For metabolomics analyses, Orthogonal Partial Least Squares-Discriminant Analysis (OPLS-DA) with permutation testing (200 iterations) was employed for multivariate discrimination. Differential metabolites were selected based on an absolute log_2_ fold change (|log_2_FC|) greater than 1 or 2, as specified in the figure legends, and a false discovery rate (FDR)-adjusted *p* < 0.05. KEGG pathway enrichment was evaluated using the hypergeometric test with FDR correction, and Spearman correlation analysis was applied to assess microbiota-metabolite associations. Sample sizes for each experiment are detailed in the respective figure legends. All statistical tests were two-sided.

## 3 Results

### 3.1 Establishing animal models of WMI

**Figure 1:**
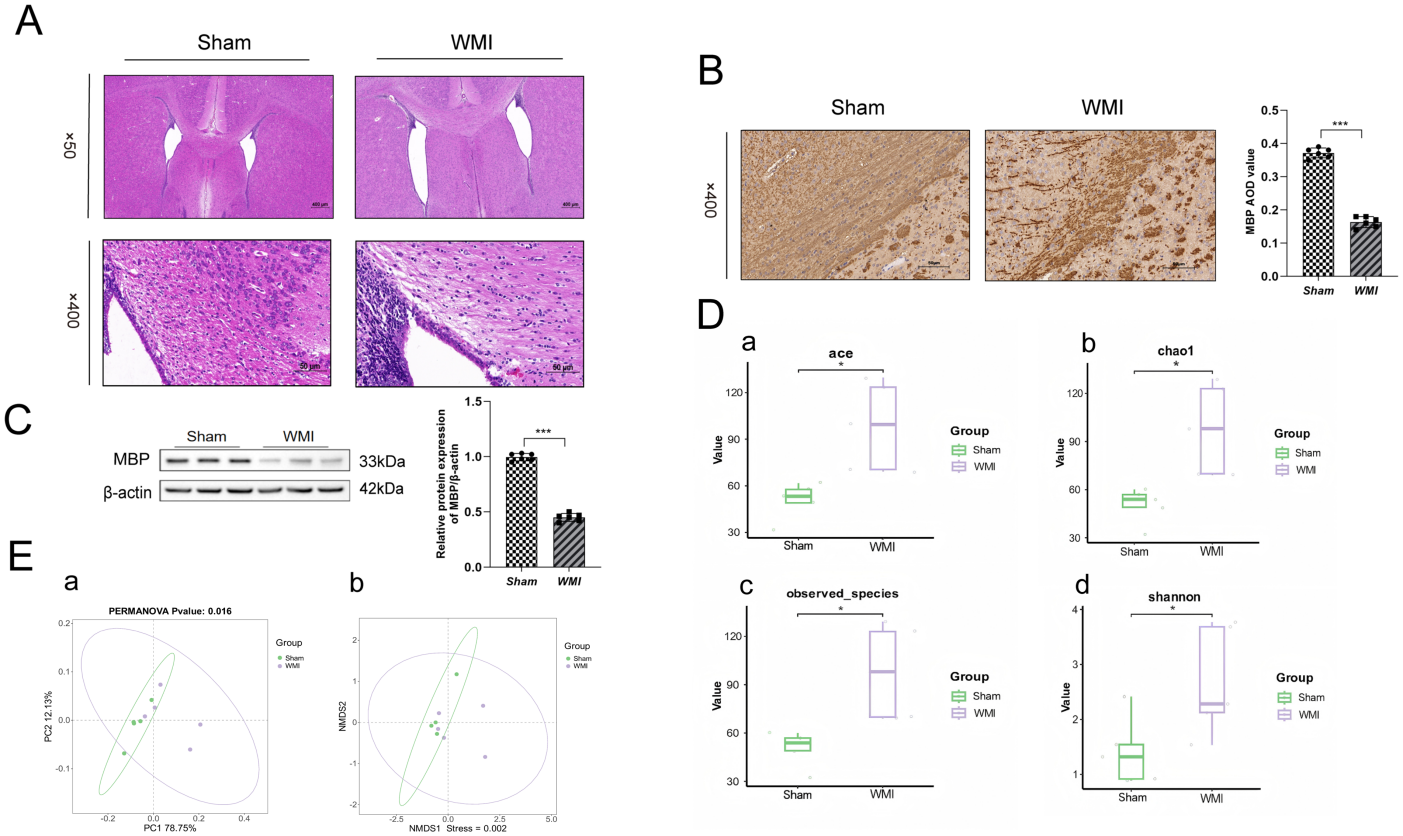
Validation of the WMI rat model and comparison of gut microbiota alpha- and beta-diversity between the WMI and Sham groups. **(A)** Representative H&E staining images showing pathological changes in the periventricular white matter of rats from the WMI and Sham groups. Scale bars: 400 μm (upper panels); 50 μm (lower panels). **(B)** Immunohistochemical staining for MBP in the left corpus callosum of rat brains from the WMI and Sham groups. Scale bar: 50 μm. **(C)** Western blot analysis of MBP protein expression levels in brain tissue from the WMI and Sham groups. **(D)** Comparison of alpha-diversity indices (Ace, Chao, Observed_species, and Shannon) between the two groups. **(E)** Principal coordinates analysis (PCoA) plot demonstrating distinct clustering of gut microbiota between the two groups. **(F)** Non-metric multidimensional scaling (NMDS) plot indicating significant separation of microbial communities. Statistical analysis: Data in **(C)** and **(D)** were analyzed using the Mann–Whitney *U* test. Intergroup differences in beta-diversity **(E,F)** were assessed by PERMANOVA. Data are presented as the mean ± SEM. **(A–C)**: *n* = 6 per group. **(D–F)**: *n* = 5 per group. ^*^p < 0.05, ^**^p < 0.01, ^***^p < 0.001.

The successful establishment of the WMI model was confirmed through histopathological and molecular analyses. Histological examination via Hematoxylin and Eosin (H&E) staining revealed that the ligated lateral ventricle was enlarged in the WMI group compared to Sham controls, with pronounced structural damage in the white matter tracts characterized by tissue rarefaction, vacuolization, and disrupted cytoarchitecture. Immunohistochemical (IHC) analysis further substantiated these findings, demonstrating a marked reduction in the immunoreactivity of myelin-related markers within the lesion areas, consistent with demyelination and axonal injury. Western blot analysis provided quantitative evidence of myelin loss. The protein expression level of Myelin Basic Protein (MBP), a critical component of the myelin sheath, was significantly lower in the WMI model group compared to the Sham group (*p* < 0.001), as shown in Figures 1A–C.

### 3.2 WMI induces alterations in gut microbiota richness and community structure

After clearly establishing the success of the model construction, the evaluation of microbial community diversity and homogeneity was carried out via alpha diversity analysis (Figure 1D). The findings indicated that the Ace, Chao1, Observed_species, and Shannon indices exhibited significant increases (*p* < 0.05), implying increased diversity and richness in the WMI relative to the Sham group. Beta-diversity analyses, specifically PCoA (based on weighted UniFrac distance) and NMDS (based on Bray-Curtis distance), demonstrated a significant separation between the WMI and Sham groups (PERMANOVA test yielded a significant result, *p* < 0.05), indicating that the overall microbial community structures are distinct between the groups (Figure 1E). Furthermore, the greater dispersion among WMI samples in the ordination plots suggests higher inter-individual variability in gut microbiota composition compared to the more homogeneous Sham group.

### 3.3 WMI is associated with a shifted gut microbiota composition at multiple taxonomic levels

**Figure 2:**
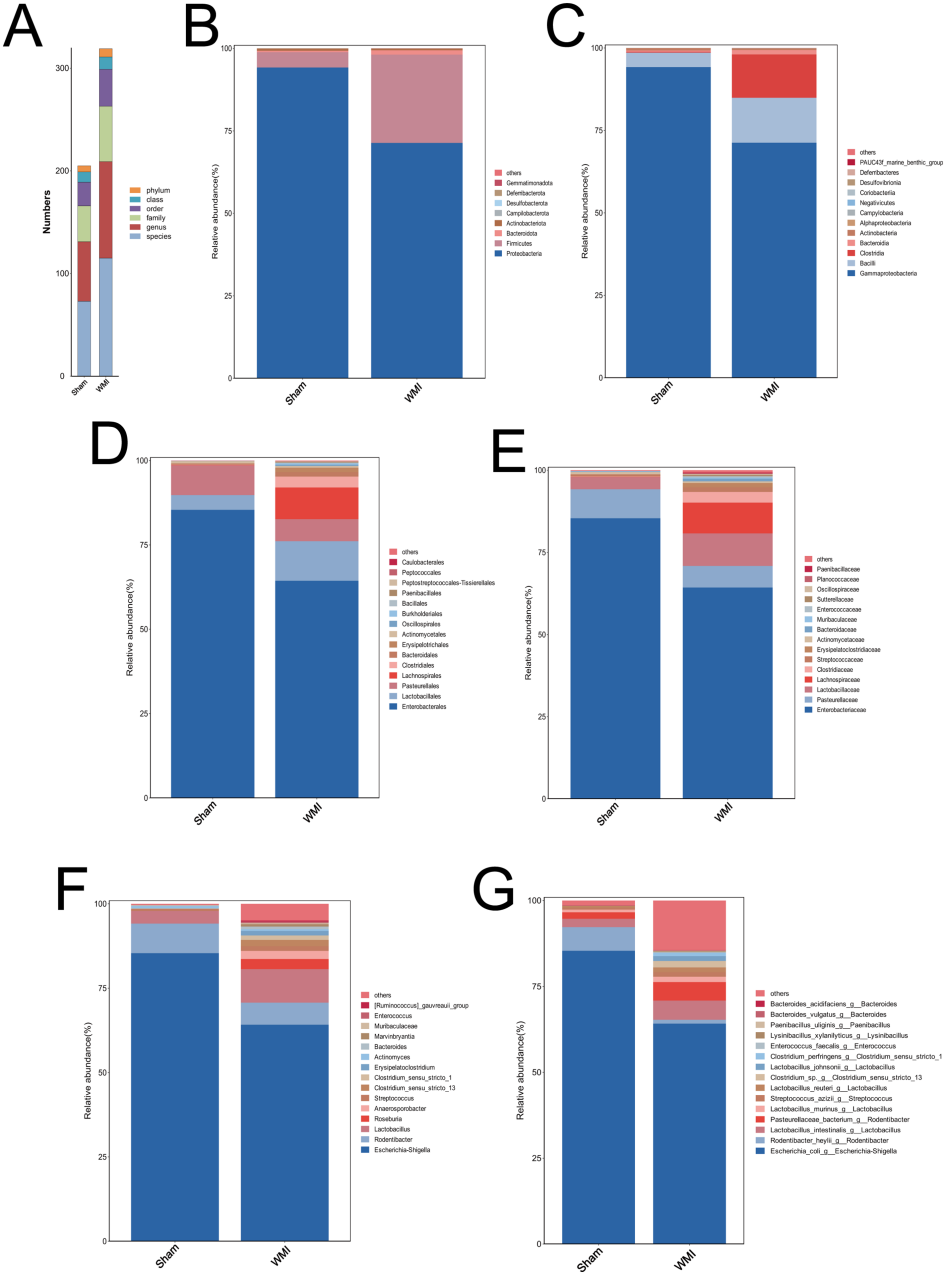
16S rRNA gene sequencing analysis of gut microbiota composition. **(A)** Bar plot showing the total number of amplicon sequence variants (ASVs) in each group. **(B–G)** Stacked bar plots displaying the relative abundance of gut microbiota at different taxonomic levels: **(B)** phylum, **(C)** class, **(D)** order, **(E)** family, **(F)** genus, and **(G)** species. Statistical analysis: data are presented as the mean ± SEM. Differences between groups were assessed using ANCOM. *n* = 5 per group (*p* < 0.05).

The gut microbiota composition was analyzed at six taxonomic levels (phylum, class, order, family, genus, and species). Statistical analysis revealed distinct compositional profiles between the WMI and Sham groups. Specifically, the WMI group exhibited significantly increased relative abundances of multiple taxa at each level (*p* < 0.05), a pattern that was reflected in the taxon count distribution (Figure 2A). Analysis of the top 15 most abundant amplicon sequence variants (ASVs) further confirmed this compositional shift, indicating a significantly more complex community structure in the WMI group (*p* < 0.05, Figures 2B–G).

### 3.4 LEfSe analysis identifies WMI-associated characteristic microbial taxa

**Figure 3:**
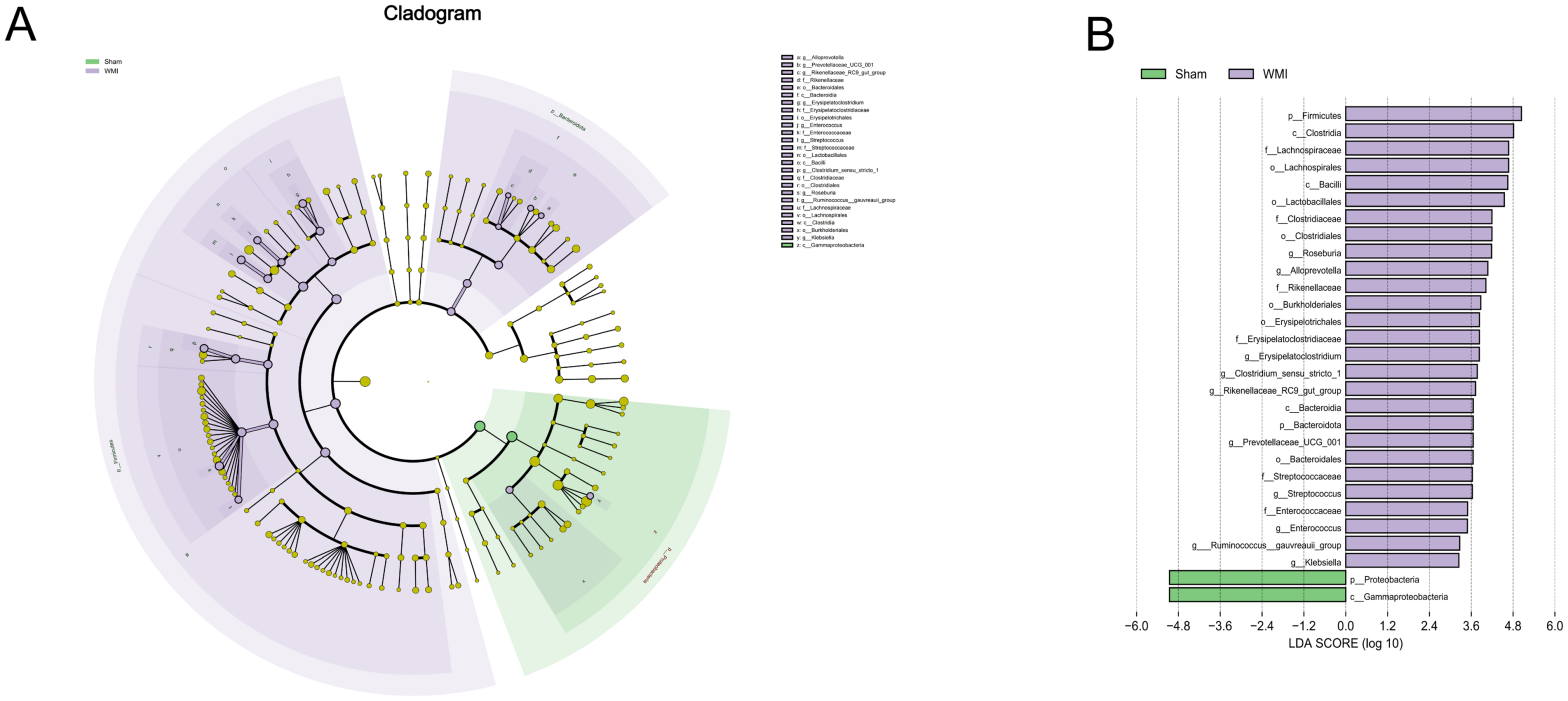
LEfSe analysis of differentially abundant gut microbiota taxa. **(A)** Cladogram from LEfSe analysis illustrating the phylogenetic distribution of microbial lineages with significant abundance differences. Green and purple areas represent taxa enriched in the Sham and WMI groups, respectively. Yellow dots indicate taxa with no significant difference. Node size reflects relative abundance. **(B)** LDA effect size (LDA score > 2.0) for differentially abundant taxa. Positive LDA scores (purple bars) indicate taxa enriched in the WMI group; negative scores (green bars) indicate taxa enriched in the Sham group. Bar length represents the magnitude of abundance difference. Statistical analysis: differentially abundant taxa were identified by Linear Discriminant Analysis Effect Size (LEfSe) analysis (LDA score > 2.0, *p* < 0.05). *n* = 5 per group.

LEfSe analysis revealed significant differences in the gut microbiota composition between the WMI and Sham groups (LDA score > 2, *p* < 0.05 after FDR correction for multiple testing). The WMI group was characterized by a marked enrichment of several taxa belonging to the phyla *Firmicutes* and *Bacteroidetes*, including the genera *Erysipelatoclostridium*, *Enterococcus*, and *Streptococcus* (Figures 3A,B). In contrast, the Sham group displayed a higher relative abundance of taxa belonging to the phylum *Proteobacteria*, notably the class *γ-Proteobacteria*. These differentially abundant microbial taxa could constitute a distinct microbial signature associated with WMI. Future studies with larger cohorts and validated predictive models are warranted to assess their potential as diagnostic or prognostic biomarkers.

### 3.5 Metabolomic profiling reveals systemic metabolic remodeling in WMI and identifies EPA as a key metabolite

**Figure 4:**
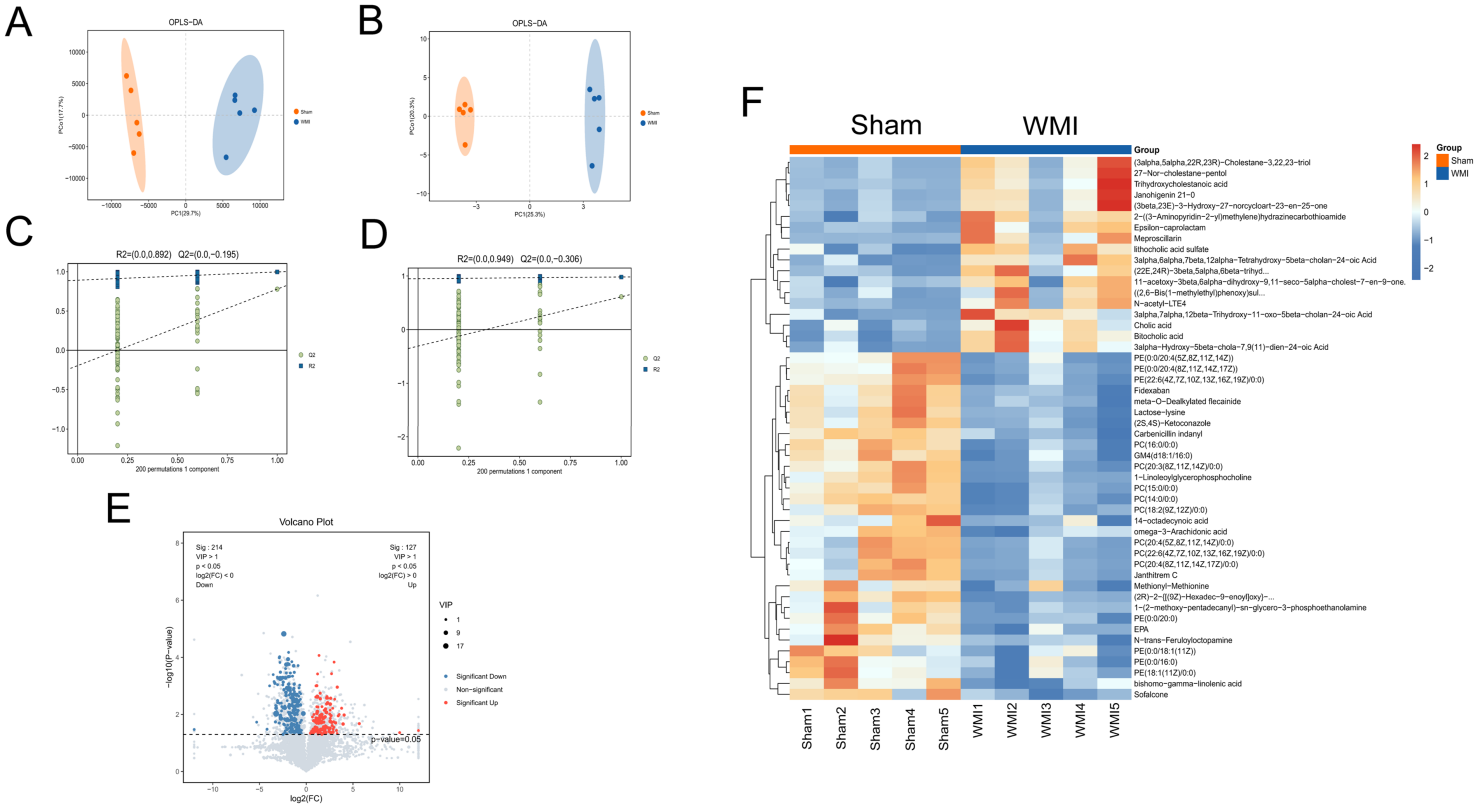
Metabolic profiling and differential metabolite analysis between Sham and WMI groups. **(A)** Orthogonal partial least squares-discriminant analysis (OPLS-DA) score plot based on liquid chromatography-mass spectrometry (LC–MS) data. **(B)** Orthogonal partial least squares-discriminant analysis (OPLS-DA) score plot based on gas chromatography–mass spectrometry (GC–MS) data. **(C,D)** Permutation tests (200 iterations) for the OPLS-DA models in **(A,B)**, respectively. Negative intercepts of Q^2^ (C: R^2^ = 0.892, Q^2^ = −0.195; D: R^2^ = 0.949, Q^2^ = −0.306) indicate no overfitting, validating model reliability. **(E)** Volcano plot displaying differentially abundant metabolites. Red dots: significantly upregulated; blue dots: significantly downregulated; gray dots: non-significant. VIP values indicate metabolite importance in group discrimination. **(F)** Heatmap of the top 50 differentially abundant metabolites, with hierarchical clustering of rows and columns. Statistical analysis: multivariate OPLS-DA was performed to distinguish metabolic profiles, with model validity assessed by permutation tests (200 iterations). Differential metabolites were identified by combining orthogonal partial least squares-discriminant analysis (OPLS-DA) VIP > 1 with unpaired *t*-tests or Mann–Whitney *U* test (depending on data distribution), and *p*-values were adjusted by the false discovery rate (FDR) method. Metabolites with |log_2_₂FC| > 1 and FDR-adjusted *p* < 0.05 were considered significant. *n* = 5 per group.

Given that gut microbiota play a crucial role in host metabolism, we subsequently performed non-targeted metabolomics to reveal systemic metabolic changes in WMI and to identify potential critical functional molecules. Metabolites with significant differential abundance were selected based on *p* values obtained from *t*-tests and variable importance in projection (VIP) scores from Orthogonal Partial Least Squares-Discriminant Analysis (OPLS-DA). OPLS-DA score plots based on liquid chromatography-mass spectrometry (LC–MS) and gas chromatography–mass spectrometry (GC–MS) data both showed clear separation between the WMI and Sham groups, indicating distinct metabolic profiles (Figures 4A,B). The stability and reliability of the OPLS-DA model were confirmed by a permutation test, which showed strong within-group correlation and significant between-group separation, validating the data quality and the existence of significant metabolic alterations (Figures 4C,D). Using thresholds of *p* < 0.05 and VIP > 1, we identified 341 differential metabolites, including 127 that were upregulated and 214 that were downregulated in the WMI group (Figure 4E).

To visualize the overall pattern of these alterations, a heatmap of the top 50 differentially abundant metabolites was generated (Figure 4F). This analysis highlighted that steroid bile acids such as cholic acid were markedly elevated in WMI (*p* < 0.05). In contrast, several neuroprotective and structural lipids were significantly downregulated (*p* < 0.05), including phosphatidylcholine (PC), eicosapentaenoic acid (EPA), phosphatidylethanolamine (PE), and *ω*-3 arachidonic acid. These distinct intestinal metabolite profiles suggest that metabolic shifts may play an important role in WMI pathogenesis. The differences in intestinal metabolites between the two groups indicated that intestinal metabolites may play an important role in the occurrence of WMI.

**Figure 5:**
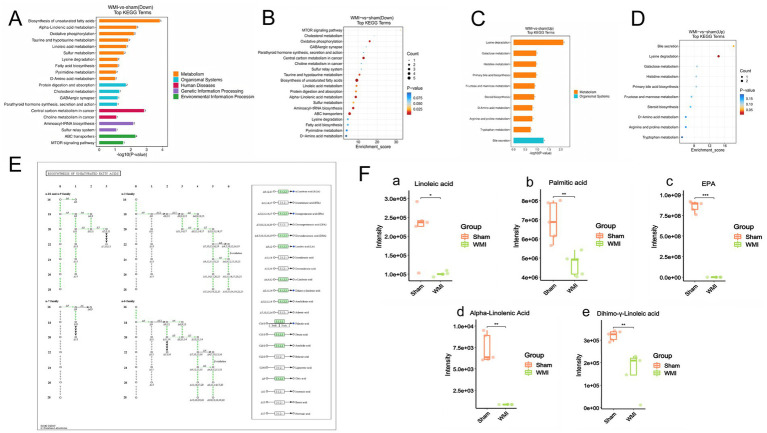
KEGG pathway enrichment analysis of differential metabolites and functional analysis of unsaturated fatty acids. **(A)** Histogram showing KEGG pathway enrichment of down-regulated differential metabolites. **(B)** Bubble plot showing KEGG pathway enrichment of down-regulated differential metabolites. **(C)** Histogram showing KEGG pathway enrichment of up-regulated differential metabolites. **(D)** Bubble plot showing KEGG pathway enrichment of up-regulated differential metabolites. **(E)** Schematic diagram of the unsaturated fatty acid biosynthesis pathway. **(F)** Boxplots showing the relative levels of representative unsaturated fatty acids in the WMI and Sham groups. Statistical analysis: Pathway enrichment analysis was performed using the hypergeometric test, with *p*-values adjusted by the FDR method. Enriched pathways were defined as those with FDR-adjusted *p* < 0.05. Data in **(F)** are presented as mean ± SEM and were compared using an unpaired *t*-test or Mann–Whitney *U* test. *n* = 5 per group. ^*^p < 0.05, ^**^p < 0.01, ^***^p < 0.001.

Downregulated metabolites were found to play significant roles in several key functional pathways, including the biosynthetic pathway of unsaturated fatty acids, ABC transporter, aminoacyl-tRNA biosynthesis, *α*-linoleic acid metabolism, and oxidative phosphorylation. Furthermore, these metabolites were enriched in the MTOR signalling pathway, GABAergic signalling pathways, cholesterol metabolism, and other related pathways (*p* < 0.05), as shown in Figures 5A,B. Among the functional pathways annotated by KEGG, upregulated metabolites were significantly involved in lysine degradation and bile secretion (*p* < 0.05), as shown in Figures 5C,D. The analysis of KEGG pathway enrichment indicated that the down-regulated metabolites were particularly significant within the unsaturated fatty acid synthesis pathway (*p* < 0.001), as shown in Figure 5E. These findings showed the probable involvement of intestinal metabolites in the MGBA in WMI.

Consequently, we analysed differentially expressed unsaturated fatty acids in fecal metabolites, identifying linolenic acid, palmitic acid, Dihimo-γ-linolenic acid, EPA, and ɑ-linolenic acid as significantly different (*p* < 0.05), as shown in Figure 5F. Among these, the reduction in the unsaturated fatty acid EPA in fecal metabolites was the most pronounced in the WMI group (*p* < 0.001). It was speculated that EPA may be a key metabolite affecting WMI.

### 3.6 Correlation network implicates characteristic gut microbiota in the depletion of protective metabolites like EPA

**Figure 6:**
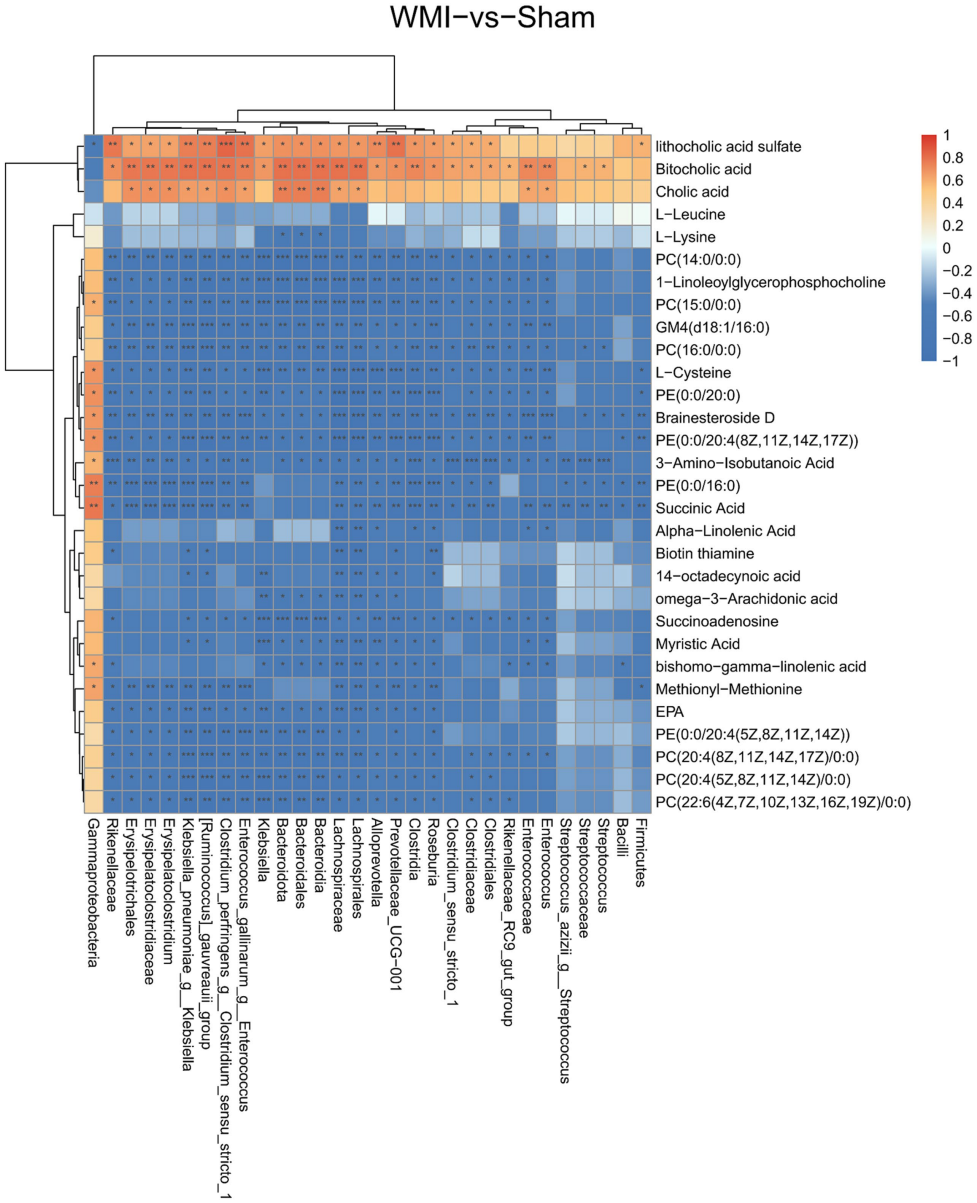
Correlation between gut microbiota and metabolites. Heatmap of Spearman correlation coefficients between genus-level gut microbiota abundance and differential metabolite levels (WMI vs. Sham). Red indicates a positive correlation; blue indicates a negative correlation. Color intensity reflects correlation strength. ^*^p < 0.05, ^**^p < 0.01, ^***^p < 0.001.

To explore the functional links between gut dysbiosis and metabolic alterations, we performed Spearman correlation analysis. Notably, a significant negative correlation was observed between WMI-enriched genera (such as *Erysipelatoclostridium* and *Klebsiella*) and the levels of several neuroprotective metabolites, most notably EPA (*p* < 0.05; Figure 6). This association implicated the characteristic gut microbiota in the observed depletion of EPA, providing a potential mechanistic link within the microbiota-gut-brain axis.

### 3.7 Fecal microbiota transplantation exerts neuroprotection by upregulating brain EPA levels

**Figure 7:**
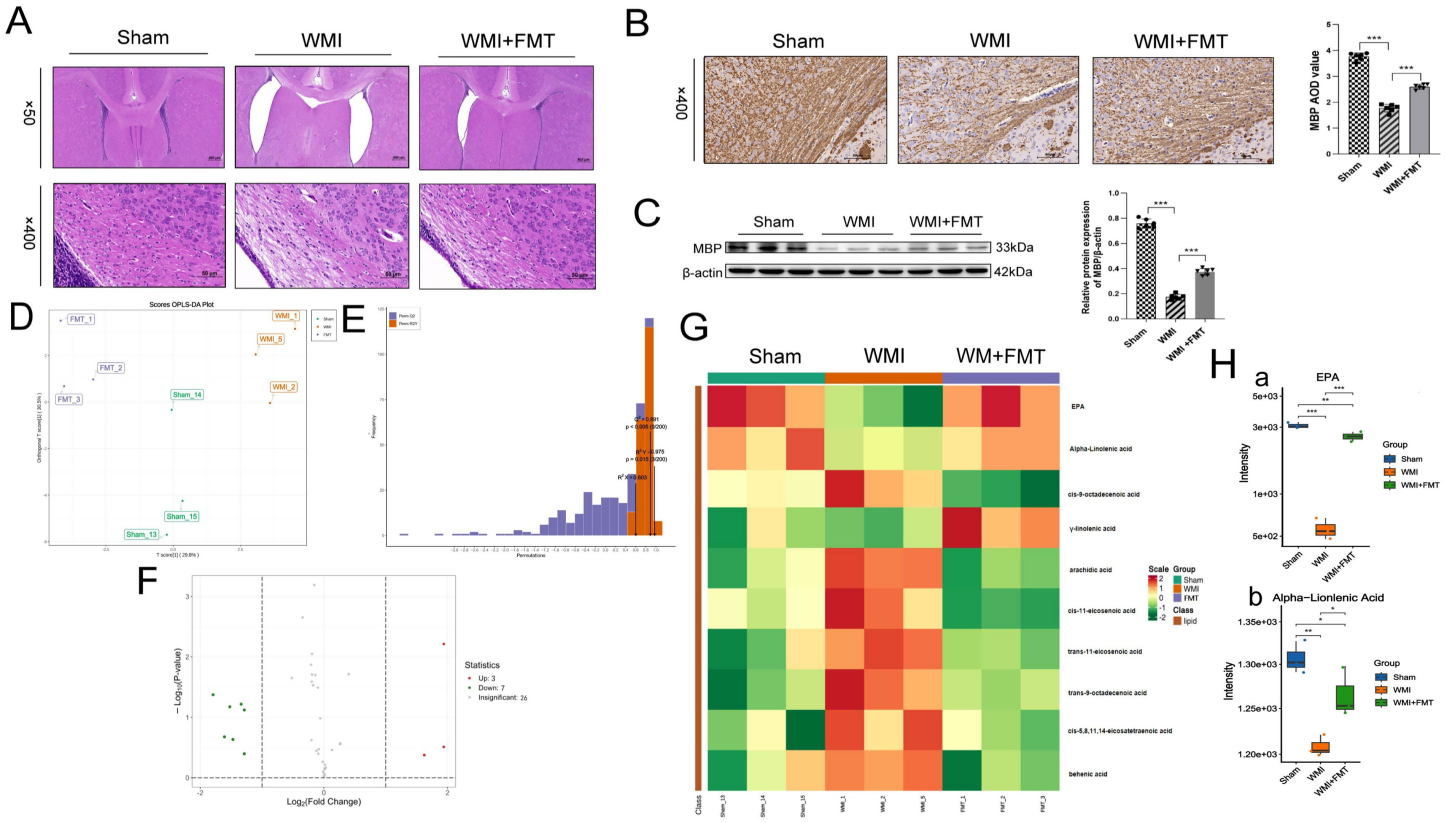
Neuroprotective effects of fecal microbiota transplantation on WMI. **(A)** Representative H&E staining images showing pathological changes in the periventricular white matter of rats. Scale bars: 400 μm (upper panels); 50 μm (lower panels). **(B)** Immunohistochemical staining for MBP in the left corpus callosum. Scale bar: 50 μm. **(C)** Protein expression levels of MBP in brain tissue, analyzed by western blot. **(D)** OPLS-DA score plot based on brain tissue metabolomics data, distinguishing metabolic profiles among the three groups. **(E)** Permutation test (200 permutations) for the OPLS-DA model in **(D)**, with Q^2^ = 0.882 and R^2^Y = 0.975. **(F)** Volcano plot visualizing differentially abundant metabolites in brain tissue among the three groups. **(G)** Heatmap of significantly differential metabolites among the three groups, with hierarchical clustering. (**H)** Analysis of unsaturated fatty acid levels in brain tissue among the three groups. Statistical analysis: Data **(C,H)** are presented as mean ± SEM and were analyzed using one-way ANOVA followed by Tukey’s post-hoc test. Multivariate OPLS-DA **(D)** was performed, with model overfitting evaluated by permutation testing **(E)**. Differential metabolites **(F,G)** were identified using one-way ANOVA (or Kruskal-Wallis test) with FDR adjustment; metabolites with |log₂FC| > 2 and FDR-adjusted *p* < 0.05 were considered significant. **(A–C)**: *n* = 6 per group. **(D–H)**: *n* = 3 per group. ^*^p < 0.05, ^**^p < 0.01, ^***^p < 0.001.

These results suggest that gut dysbiosis and brain EPA deficiency are potentially associated with the pathological process of WMI. To verify the causal relationship, we performed fecal microbiota transplantation and evaluated its effects on brain tissue metabolites and pathological changes. HE staining showed normal periventricular white matter tissue structure of the Sham group, with neatly arranged cells and a typical morphology. In the WMI group, the ligated lateral ventricle was enlarged, accompanied by a marked reduction or loss of periventricular cells, which appeared loosely and irregularly arranged. Some nuclei exhibited pyknotic changes. In the FMT group, fecal microbiota transplantation ameliorated ventricular enlargement, increased the number of periventricular cells, and improved cellular organization, indicating that FMT attenuated pathological damage caused by hypoxic–ischemic WMI (Figure 7A).

IHC revealed that FMT increased the density of MBP-positive myelinated nerve fibres in the corpus callosum of WMI rats (Figure 7B), indicating that FMT promoted recovery from myelin development disorders following WMI. Consistently, Western blotting revealed significantly higher expression of MBP in the FMT compared with WMI groups (*p* < 0.001; Figure 7C).

Multidimensional statistical analysis of targeted metabolomics with the OPLS-DA model and permutation test confirmed that the model was reliable and stable. Repeated samples within each group showed a strong correlation, with significant differences between groups, revealing reliable and significant variations in metabolites between the three groups (Figures 7D,E). Utilizing *p* values from *t*-tests and VIP values obtained from the OPLS-DA model, we selected metabolites that exhibited significant distinctions. A threshold of *p* < 0.05 and VIP > 1 was established to denote significant differences among the metabolites. Ten potential metabolic markers were identified in the three groups, including three up- and seven down-regulated metabolites (Figure 7F).

We conducted a comparative analysis of the specific fatty acid concentrations within the brain tissues across the three distinct groups. In the WMI group, metabolites like *α*-linolenic acid and EPA were down-regulated (*p* < 0.05), while arachidic acid was up-regulated (*p* < 0.05). In the FMT group, α-linolenic acid and EPA were upregulated (*p* < 0.05), whereas arachidic acid was downregulated (*p* < 0.05), as shown in Figure 7G. The changes in α-linolenic acid and EPA concentrations in brain tissue were synchronised with those in the feces. Subsequent examination revealed that the alteration in the EPA concentration was particularly significant (*p* < 0.001; Figure 7H). These findings suggested that fecal microbiota transplantation can upregulate EPA concentrations in the brain tissue and play a neuroprotective role, with EPA possibly playing a key protective role.

### 3.8 Exogenous EPA supplementation promotes remyelination and alleviates neuroinflammation via the BDNF/TrkB pathway

**Figure 8:**
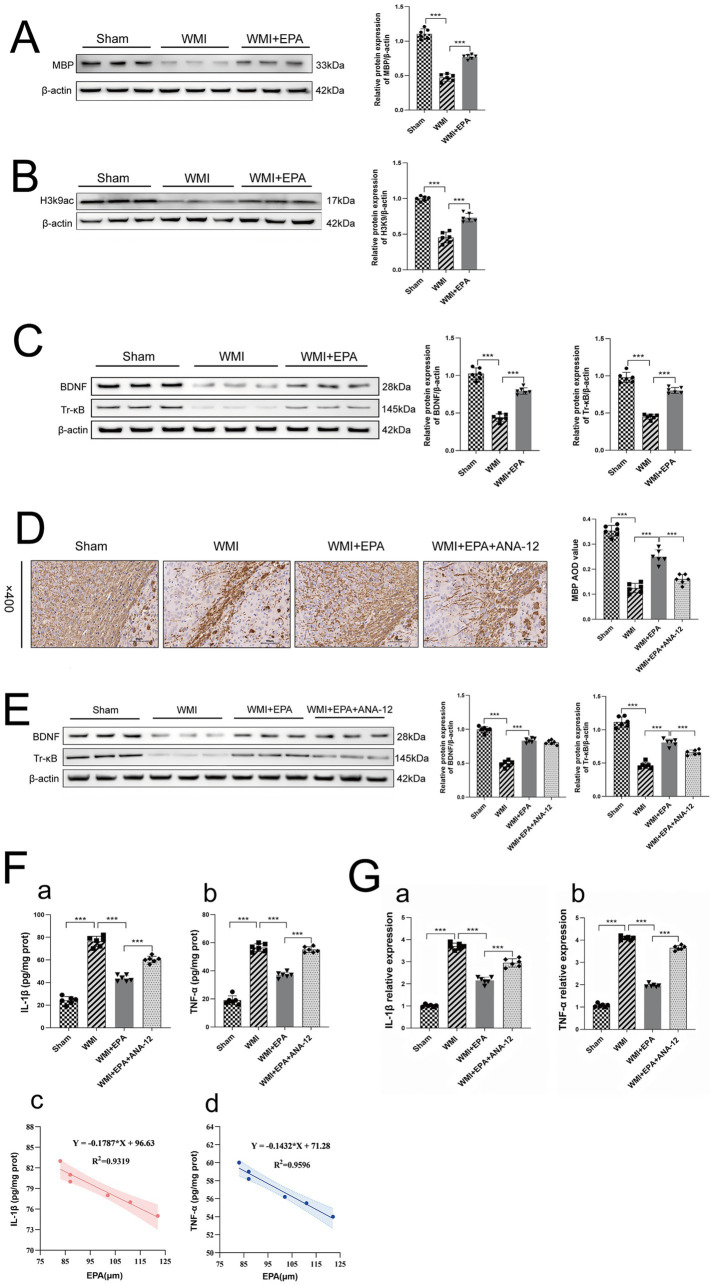
Neuroprotective effects of EPA on WMI and related mechanisms. **(A)** Protein expression levels of MBP in brain tissue from three groups, analyzed by Western blot. **(B)** Protein expression levels of H3K9ac in brain tissue from three groups, analyzed by Western blot. **(C)** Protein expression levels of BDNF/TrkB in brain tissue from three groups, analyzed by Western blot. **(D)** Immunohistochemical staining for MBP in the left corpus callosum of rat brain from four groups. **(E)** Protein expression levels of BDNF/TrkB in brain tissue from four groups, analyzed by Western blot. **(F)** Protein levels of **(a)** IL-1β and **(b)** TNF-*α* in brain tissue from four groups, analyzed by ELISA. Correlation analysis between EPA levels and **(c)** IL-1β or **(d)** TNF-α in brain tissue of WMI rats. **(G)** mRNA expression levels of (a) IL-1β and (b) TNF-α in brain tisue from four groups. Statistical analysis: Data are presented as mean ± SEM and were analyzed using one-way ANOVA followed by Tukey’s post-hoc test **(A–G)**. Correlation analyses in **F (c,d)** were performed using Spearman correlation coefficient. *n* = 6 per group. ^*^p < 0.05, ^**^p < 0.01, ^***^p < 0.001.

To directly demonstrate the neuroprotective effect of EPA and elucidate the downstream mechanism of EPA, we administered exogenous EPA to WMI rats. According to Western blotting, the EPA group’s MBP expression was significantly greater than that of the WMI group (*p* < 0.001), whereas the MBP expression within the WMI group was markedly diminished compared to that observed in the Sham group (*p* < 0.001), as shown in Figure 8A. This suggests that EPA improves recovery from myelin developmental problems linked to WMI. H3 lysine 9 acetylation (H3K9ac) plays a role in epigenetic control of cognitive and memory-related genes (Zhou et al., 2021). Western blot examination demonstrated that the WMI group had lower expression of acetylated histone H3K9ac than the Sham group, whereas the EPA intervention boosted it compared to the WMI group (*p* < 0.001), as shown in Figure 8B. Histone acetylation protects oligodendrocytes from WMI through activation of the BDNF/TrkB signaling pathway in immature brains (Huang et al., 2018). The BDNF/TrkB pathway downstream of acetylated histones was identified using Western blot in the three groups of rats. The findings indicated that the WMI group exhibited reduced levels of BDNF and TrkB expression compared to the Sham group (*p* < 0.001), a difference that was mitigated by EPA intervention (*p* < 0.001), as shown in Figure 8C. This was in line with the levels of acetylated histone H3K9ac. These results indicated that EPA promoted histone acetylation and enhanced the stimulation of the downstream BDNF/TrkB pathway.

The analysis of MBP through IHC in the left corpus callosum revealed that both MBP and myelinated nerve fibre densities were elevated in the WMI + EPA group compared to the WMI group (*p* < 0.001), while a reduction was observed in the WMI + EPA + ANA-12 group compared to the WMI + EPA group (*p* < 0.001), as shown in Figure 8D. This suggests that EPA helped WMI recover from myelin development problem, while the TrkB inhibitor ANA-12 lessened this effect. The expression levels of BDNF/TrkB were assessed in each of the four rat groups utilizing the western blot technique. Following EPA intervention, BDNF expression was greater than in the WMI group (*p* < 0.001); however, there was no difference in the WMI + EPA + ANA-12 group (*p* > 0.05). TrkB was higher after EPA intervention than in the WMI group (*p* < 0.001), but lower in the WMI + EPA + ANA-12 group than the EPA group (*p* < 0.001), as shown in Figure 8E. The findings substantiated that the TrkB inhibitor ANA-12 markedly diminished the stimulation of the BDNF/TrkB pathway prompted by EPA.

The ELISA immunosorbent test measured inflammatory agents in all four rat groups. The WMI group had significantly higher levels of TNF-*α* and IL-1*β* relative to the Sham group (*p* < 0.001). After EPA intervention, TNF-α and IL-1β levels were significantly lower in the WMI + EPA group (*p* < 0.001), while the WMI + EPA group had lower levels than the WMI + EPA + ANA-12 group (*p* < 0.001), as shown in Figure 8F. In the WMI model, analysis of brain tissue demonstrated strong negative linear relationships between EPA concentration and the expression of both TNF-α (R^2^ = 0.9596; Y = −0.1432X + 71.28) and IL-1β (R^2^ = 0.9319; Y = −0.1787X + 96.63), suggesting that a reduction in EPA levels is strongly correlated with elevated inflammatory responses in the injured brain (Figure 8F). The WMI group had higher TNF-α and IL-1β mRNA levels than the Sham group (*p* < 0.001). The WMI + EPA + ANA-12 group had higher IL-1β and TNF-α mRNA levels than the WMI + EPA group (*p* < 0.001), while the WMI + EPA group had lower levels than the WMI group (*p* < 0.001), as shown in Figure 8G. TrkB inhibitor ANA-12 reversed EPA-induced reduction of periventricular white matter inflammation.

## 4 Discussion

By altering the metabolome, transcriptome, and epigenome, the gut microbiome regulates host health. This study used 16S rRNA gene sequencing and LC–MS/GC–MS dual-platform metabolomics to examine the gut microbiome and metabolite changes in rats with hypoxic-ischemia WMI and their putative relationship. This study demonstrated notable variations between the WMI and Sham groups as well as a strong link between the gut microbiota and its metabolites, therefore suggesting the possible role of gut microbiome metabolites in WMI pathogenesis. Overall, this study demonstrates that WMI is associated with a significant gut dysbiosis, highlights the importance of the gut microbiome in WMI and provides fresh ideas for the prediction of WMI biomarkers. Increasing evidence suggests that gut microbes are associated with brain and behavioural outcomes. Our study found significant variations in the structure, content, and function of the gut microbiota across WMI and Sham groups, at the class, family, genus, order, phylum, and species levels, including in *Erysipelatoclostridiaceae*, *Klebsiella*, *Streptococcus*, *Rikenellaceae*, *Clostridium*, and *Enterococcus*. Gut microbiota abundance was significantly greater in the WMI group compared to the Sham group, indicating a potential pathogenic connection. This agrees with prior studies. One study found that fecal samples of preterm newborns were considerably enriched with *Klebsiella* within 2 weeks, while full-term newborns exhibited a rise in *Klebsiella* after 6 months (Yap et al., 2021). In particular, *Klebsiella* is connected with pro-inflammatory responses, and its expansion in the gastrointestinal system is a powerful predictor of brain damage (Seki et al., 2021). According to another study, babies born prematurely with WMI had a higher abundance of *Staphylococcus* species, such as *S. caprae*, as well as *Bacteroidetes*, *Actinobacteria*, and *Acinetobacter* species (Liu et al., 2023). The phylum *Bacteroidetes* represents the most extensive assemblage of gram-negative bacteria found within the gastrointestinal microbiota. While *Bacteroidetes* are favorable to the host when restricted to the gastrointestinal tract, they can injure other bodily regions by secreting pro-inflammatory neurotoxins, such as lipopolysaccharides, and toxic proteolytic metabolites, causing nervous system injury (Zhao and Lukiw, 2018). *Bacteroides* plays a role in the progression of Alzheimer’s disease (AD) by impairing microglial phagocytosis, thereby reducing amyloid β clearance, leading to amyloid plaque accumulation (Wasén et al., 2024). Infants with autism spectrum disorders have a higher prevalence of *Clostridia* and *Klebsiella* species in their intestines (Zuffa et al., 2023), while *Lachnospira* is very common in the guts of infants with behavioural problems. *Clostridium* species have been linked to suboptimal cognitive growth and behavioral issues. A higher abundance of *Clostridiumsensustricto1* is negatively connected to the ASQ-3 communication score (Zhang et al., 2021). A fecal microbiota dominated by *Klebsiella* and *Enterococcus* is connected with a higher prevalence of NEC in prematurely born babies (McCartney and Hoyles, 2023). Based on LEfSe analysis, we postulate *Klebsiella*, *Lachnospiraceae*, *Clostridium_sensu_stricto_1*, *Erysipelatoclostridiaceae, Rikenellaceae, Enterococcus, Streptococcus,* and *Bacteroidales* as potential biomarkers for WMI. Our study found the characteristic gut microbiota of WMI.

The MGBA is a multidimensional network that allows brain-gut communication. Central, autonomic, enteric, neuroendocrine, and neuroimmune systems are included in this framework (Filpa et al., 2016; Sánchez et al., 2017). Through control of motility, secretion, nutritional absorption, and immunological responses, this dual route lets the CNS sustain and control digestive processes (Lee et al., 2023; Naufel et al., 2023). In contrast, signals originating from the intestines can impact the progression of the CNS. The initial phases of life are crucial for brain development and development, during which nutritional strategies, including the administration of probiotics, prebiotics, engineered bacteria, and maternal dietary practices, can exert beneficial effects on gut-brain signalling and facilitate brain development (Ratsika et al., 2021).

Besides maintaining gastrointestinal homeostasis, the gut microbiota and its metabolites regulate distant organs including the brain (Lee et al., 2023). Intestinal microorganisms produce metabolites that enter the bloodstream and traverse the membrane between the blood and the brain to exert impact on the CNS. Some intestinal microbial metabolites, like LPS, short-chain fatty acids, trimethylamine, vitamins, and unsaturated fatty acids, can have a direct impact on neuronal activity or activate the immune and endocrine systems (Zhang et al., 2022). Sodium butyrate influences the expression of neurotrophic-related genes via histone crotonylation. Altering the intestinal microbiota and levels of SCFAs in the brain may mitigate damage from hypoxic–ischemic encephalopathy (He et al., 2022), representing a novel microbial method for the prevention and treatment of hypoxic-ischaemic encephalopathy. *Staphylococcus* may influence WMI by reducing the levels of metabolites. In addition, gut microbiota, including *Acinetobacter* and *Bacteroide*s, has the potential to alter the structure of white matter through the upregulation of metabolites, like toad venom protein (Liu et al., 2023). Indeed, changes in *Klebsiella* abundance in the intestinal tract of premature infants with WMI promote an inflammatory response that could influence cognitive processes through the regulation of SCFAs.

The findings suggest that intestinal metabolites generated by the gut microbiota could participate in the progression of WMI in premature infants. We identified notable distinctions in fecal microbial metabolites between the WMI and Sham groups, with an aggregate of 341 differential metabolites—127 exhibiting up-regulation and 214 down-regulation in the WMI group. A considerable number of these metabolites were associated with neural functional pathways, especially in relation to the synthesis of unsaturated fatty acids. Among the downregulated differential metabolites of unsaturated fatty acids, EPA emerged as the most important, highlighting its role as a crucial metabolite influencing WMI. A correlation analysis of differential gut microbiota and intestinal metabolites was conducted, uncovering that *Klebsiella, Streptococcus, Clostridium,* and *Lachnospira* could impact WMI by down-regulating certain metabolites, like EPA, *ω*-3-arachidonic acid, succinic acid, ganglioside, phosphatidylcholine, and *α*-linolenic acid, while simultaneously up-regulating cholic acid. This study found that intestinal metabolites, particularly unsaturated fatty acids, may influence WMI, presenting a novel finding that contrasts with prior research showing that intestinal metabolites contribute significantly to the advancement of WMI. As such, we investigated how the intestinal flora could mitigate the neuroinflammatory response associated with WMI through unsaturated fatty acids, thereby clarifying the role of the MGBA in WMI pathogenesis. Our results indicated that intestinal dysbacteriosis may play a role in hypoxic–ischaemic WMI by downregulating the intestinal metabolite EPA, which seems to be fundamental in the progression of white matter damage.

Fecal microbiota transplantation surfaced as a noteworthy treatment option for various neurological disorders (Vendrik et al., 2020; Antushevich, 2020). Herein, we observed a neuroprotective effect of FMT against WMI, including reduced neuroinflammation and improved myelination deficits following ischemia and hypoxia. This neuroprotective mechanism was linked to increased levels of the intestinal metabolite EPA, which affects histone acetylation in oligodendrocytes.

Many lipid derivatives are significantly altered during brain injury, ischemia, and inflammation (Bazinet and Layé, 2014; Uauy and Mena, 2015). Among these, ω-3 and ω-6 polyunsaturated fatty acids control neuroinflammation through direct action or their metabolites, affecting brain development (Martinat et al., 2021). ω-3 polyunsaturated fatty acids, including DHA, EPA, and *α*-linolenic acid, also contribute significantly to the regulation of microglia-dependent neuroinflammation. Recent research has demonstrated that sodium butyrate mitigates hypoxic–ischemic brain injury in neonatal rats through the mediation of histone crotonylation via the MGBA (He et al., 2022).

A third stage of hypoxic–ischaemic nerve injury has been identified, characterised by the continuous release of detrimental factors, such as neuroinflammatory agents and cytokines, as well as epigenetic alterations that could impede axon growth, synaptogenesis, and neurogenesis (Fleiss et al., 2021). Investigating how epigenetics interact with nerve injury repair can help understand neonatal brain injury. Previous study indicated that EPA alleviates chronic brain inflammation by potently enhancing the BDNF/TrkB neurotrophic pathway through a synergistic, multi-targeted approach. EPA’s potent anti-inflammatory action—mediated via GPR120 receptor agonism and NF-κB inhibition—directly suppresses pro-inflammatory cytokines like IL-1β and TNF-α, which are known repressors of BDNF gene expression (Oh et al., 2010). By removing this suppression, EPA upregulates BDNF synthesis and release. Concurrently, EPA incorporates into neuronal membranes, increasing fluidity and optimizing lipid raft composition for efficient TrkB receptor clustering and signaling (Suzuki et al., 2004). Enhanced BDNF binding to TrkB robustly activates downstream survival (PI3K/Akt) and plasticity (MAPK/ERK) pathways, which promote neuronal repair, synaptic integrity, and the production of anti-inflammatory mediators (Tong et al., 2008). However, it is not yet clear through which intermediaries EPA regulates the BDNF/TrkB signaling pathway.

Histone acetylation represents the most foundational and thoroughly examined type of histone modification. This process involves adding or removing acetyl groups from histone proteins, which then affects chromatin structure and, in turn, influences cellular functions and characteristics. Furthermore, it is instrumental in modulating chromatin configuration and gene expression (Sakharkar et al., 2016). Butyrate, the main metabolite of intestinal butyric acid bacteria, can reduce oxidative stress factors in the brain, inflammation, nerve apoptosis; and improve nerve cell function, mainly through BDNF-mediated action of PI3K (Liu et al., 2015). Butyrate functions as an HDAC inhibitor, demonstrating the capability to enhance spatial learning and memory, as well as promoting neuronal activity. Additionally, it has been shown to impede neuronal apoptosis in cases of hypoxic-ischaemic encephalopathy, thereby contributing to its neuroprotective effects (Kim et al., 2009). Epigenetics and metabolomics interact and regulate each other via histone acetylation. H3K9ac is enriched in genes involved in gluconeogenesis and fat metabolism and promotes transcription (Morral et al., 2021). The current investigation demonstrates that EPA contributes to neuroprotection by enhancing the levels of acetylated histone H3K9ac, which in turn improves myelination disorders and contributes to its neuroprotective effects. Increased acetylation can promote the differentiation and maturation of oligodendrocytes and improve myelination disorders, potentially via the BDNF/TrkB pathway.

BDNF serves as a crucial neurotrophic factor, significantly influencing the regulation of diverse neuronal functions. The functions encompass neuronal survival, synaptic transmission, and the long-term potentiation observed in the hippocampus, all of which are essential for sustaining memory and learning processes (Han and Holtzman, 2000; Liu et al., 2019). Additionally, BDNF exhibits neuroprotective properties against ischemic injuries by antagonizing excitatory amino acids and mitigating inflammatory responses. The stimulation of the BDNF/TrkB signalling pathway holds significant importance in the sustenance and distinction of precursor oligodendrocytes, enhancing myelin protein synthesis, influencing the thickness of the myelin sheath, and aiding in the repair of white matter injuries (Chen et al., 2018). Omega-3 polyunsaturated fatty acids regulate microglial polarization and reduce the inflammatory response after experimental traumatic brain damage via SIRT1-mediated deacetylation of the HMGB1/NF-κB pathway, thus enhancing their neuroprotective effects (Chen et al., 2023). Moreover, sodium butyrate, a metabolite derived from intestinal bacteria, has been shown to mitigate lead-induced cognitive and memory deficits via the ACSS2/H3K9ac/BDNF signalling pathway (Li et al., 2024). Unsaturated fatty acids enhance acetyltransferase activity and promote protein acetylation, improving lipid metabolism and the anti-inflammatory response.

**Figure 9:**
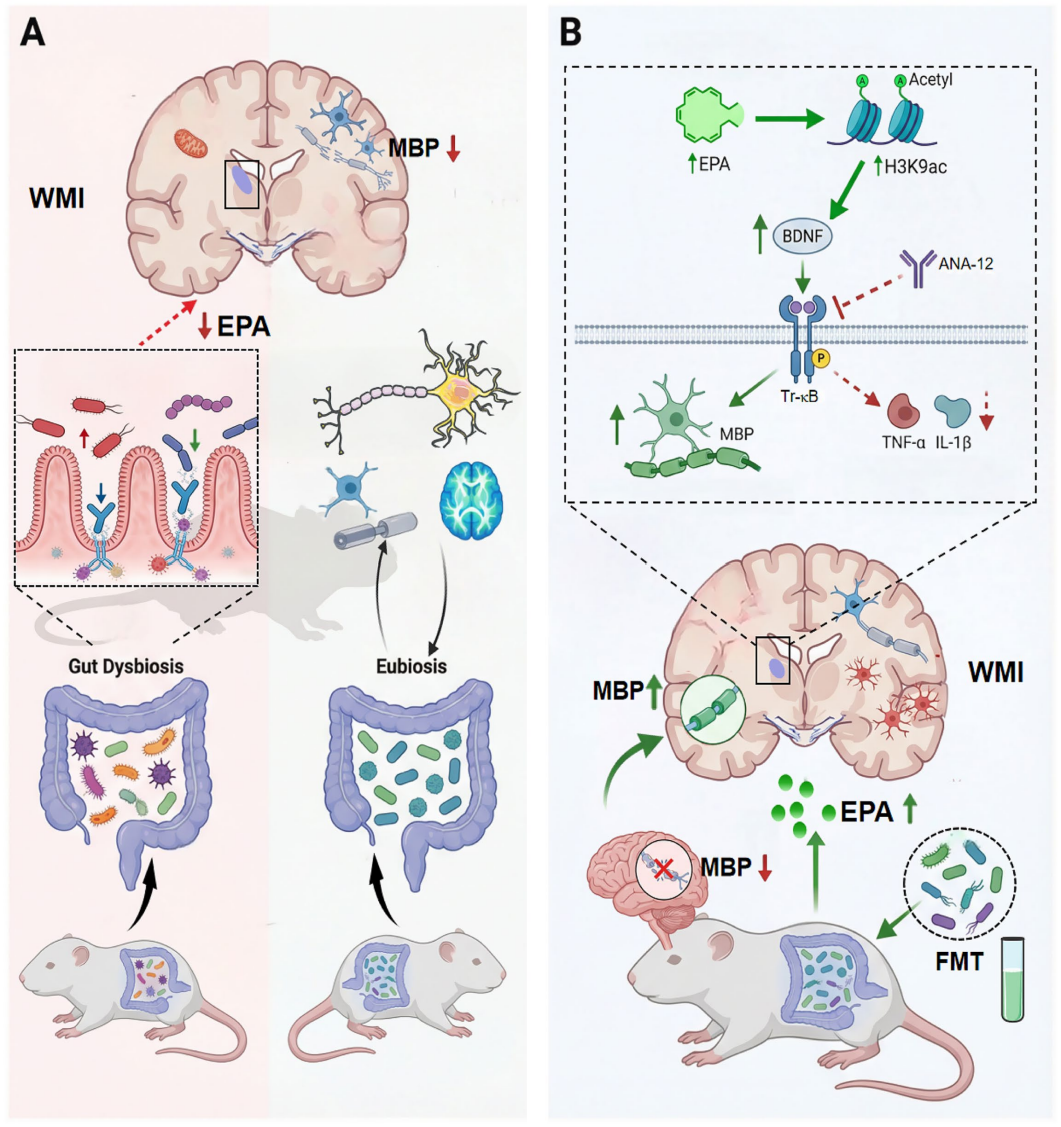
Schematic illustrating the gut-brain axis mechanism by which gut microbiota-derived EPA mediates protection against WMI. **(A)** In white matter injury, gut dysbiosis reduces EPA levels in brain tissue, leading to downregulated expression of MBP and subsequent myelin damage. In contrast, a healthy gut microbiota (eubiosis) maintains normal physiological EPA levels and supports myelin integrity. **(B)** Elevating brain EPA levels through fecal microbiota transplantation (FMT) activates the H3K9ac/BDNF/TrkB signaling pathway, which promotes MBP expression, facilitates myelin repair, and inhibits the production of pro-inflammatory cytokines (TNF-α, IL-1β). The TrkB antagonist ANA-12 blocks this protective effect. Arrow definitions: green arrows indicate an increase in levels or a protective effect; red arrows indicate a decrease in levels or an inhibitory effect; black arrows indicate physiological regulatory relationships.

This study found that after ischaemia and hypoxia, the levels of acetylated histone H3K9ac, BDNF, and TrkB protein decreased, which was rescued by EPA supplementation. The application of the TrkB receptor inhibitor ANA-12 significantly reduced TrkB expression levels. Accordingly, we speculate that the improvement in myelination disorders due to increased H3K9ac levels induced by the intestinal metabolite EPA is mediated by the BDNF/TrkB pathway. After blocking the activation of TrkB by ANA-12, although BDNF expression did not change significantly, decreased TrkB resulted in a reduction of the binding of BDNF, leading to insufficient BDNF/TrkB pathway activation to exert biological effects, eventually yielding insufficient myelinated protein synthesis. Similarly, Jaworska and Liu also found that HDACs inhibitors can activate the BDNF/TrkB pathway, promote nerve cell regeneration, and improve the prognosis of brain injury in a neonatal rat brain injury model (Kim et al., 2009). BDNF is associated with neuroinflammation in brain diseases (Lima Giacobbo et al., 2019). This study found that supplementation of hypoxic–ischaemic WMI rats with EPA significantly reduced the neuroinflammatory response and increased myelination in the periventricular white matter. ANA-12 reduced the neuroprotective effect of EPA, indicating that the neuroprotective mechanism of FMT reduces neuroinflammation by up-regulating EPA, promoting H3K9ac/BDNF/TrkB pathway activation (Figure 9).

This study represents a significant advancement as it substantiates our earlier Mendelian randomization findings that explore the causal link in gut microbiota and negative neurological development in preterm babies. Furthermore, we conducted a screening for metabolites that could significantly influence WMI via alterations in the gut microbiota and metabolome. Eventually, we investigated the processes by which gut microbiota affects the brain, identifying significant signalling pathways and molecules implicated in gut-brain interactions in WMI. This study is subject to several limitations. First, the relatively small sample size may have limited the statistical power of our analyses. Second, the findings, derived from preclinical rodent models, require validation in clinical populations to confirm their translational relevance to human neonates. Third, our mechanistic insights are preliminary. The evidence supporting the role of histone H3K9 acetylation (H3K9ac) is primarily correlative, based on the co-variation of global H3K9ac levels with downstream outcomes. We lack direct causal evidence, such as site-specific acetylome analysis (e.g., at the promoters of key genes like BDNF) or perturbation studies using pharmacological inhibitors or genetic tools to modulate H3K9ac. Consequently, the precise relationship between H3K9ac and the BDNF/TrkB pathway remains to be fully elucidated. Future research must determine whether gut microbiota-derived EPA regulates histone acetylation directly or indirectly via HDACs/HATs, and whether EPA affects white matter injury through metabolic reprogramming. Finally, the generalizability of our conclusions is constrained by the specific experimental model used.

## 5 Conclusion

The current investigation indicates that significant gut dysbiosis manifests in WMI rats, mediated by mechanisms that involve the suppression of the unsaturated fatty acid synthesis pathway. Fecal microbiota transplantation may elevate EPA levels, enhance acetylated histone H3K9ac levels, activate the BDNF/TrkB pathway, reduce the neuroinflammatory response, and ultimately improve myelination disorders in WMI. The findings elucidate a novel mechanism for WMI through the lens of gut microbiota, metabolism, and epigenetics, offering a promising avenue for therapeutic intervention in WMI.

## Data Availability

The data presented in the study are deposited in the NCBI Sequence Read Archive (SRA), accession number PRJNA1431427.

## Glossary

ASV: amplicon sequence variants
ANCOM: Analysis of Composition of Microbiomes
BDNF: Brain-Derived Neurotrophic Factor
DAMs: differentially abundant metabolites
EPA: eicosapentaenoic acid
FMT: fecal microbiota transplantation
GS: gas chromatography
HATs: histone acetyltransferases
HDACs: histone deacetylases
HE: haematoxylin and eosin
H3K9ac: histone H3 lysine 9 acetylation
HMDB: Human Metabolome Database
IHC: immunohistochemistry
IL-1β: interleukin-1β
KEGG: Kyoto Encyclopedia of Genes and Genomes
LDA: Linear discriminant analysis
LEfSe: Linear discriminant analysis effect size
LPS: lipopolysaccharides
LC–MS: liquid chromatography-mass spectrometry
MBP: myelin basic protein
MGBA: microbiome-gut-brain axis
NMDS: non-metric multidimensional scaling
OPLS-DA: orthogonal partial least squares-discriminant analysis
PC: phosphatidylcholine
PCoA: Principal coordinates analysis
PE: phosphatidylethanolamine
SCFAs: short-chain fatty acids
TrkB: tropomyosin receptor kinase B
TNF-α: tumor necrosis factor-α
VIP: variable importance in the projection
WMI: white matter injury.
